# Virtual Reality–Based Pain Modulation in Subacute Musculoskeletal Injury: Functional Near-Infrared Spectroscopy Study of Neural and Behavioral Correlates

**DOI:** 10.2196/77713

**Published:** 2026-03-30

**Authors:** Ryan Andrew Mace, Ziyan Wu, Christine B Sieberg, Keerthana Deepti Karunakaran, Ke Peng, David Borsook

**Affiliations:** 1 Center for Health Outcomes and Interdisciplinary Research, Massachusetts General Hospital, 1 Bowdoin Square Suite 100, Boston, MA, 02114, United States, 1 617-724-7030; 2 Department of Psychiatry, Harvard Medical School, Boston, MA, United States; 3 Department of Psychiatry, Massachusetts General Hospital, Boston, MA, United States; 4 Division of Adolescent and Young Adult Medicine, Boston Children's Hospital, Boston, MA, United States; 5 Athinoula A. Martinos Center for Biomedical Imaging, Department of Radiology, Massachusetts General Hospital, Charlestown, MA, United States; 6 Department of Electrical and Computer Engineering, Price Faculty of Engineering, University of Manitoba, Winnipeg, MB, Canada; 7 Department of Radiology, Massachusetts General Hospital, Boston, MA, United States

**Keywords:** immersive virtual reality, musculoskeletal pain, functional near-infrared spectroscopy, prefrontal cortex, pain management, mindfulness

## Abstract

**Background:**

Acute traumatic musculoskeletal injuries often result in persistent pain and disability despite physical recovery. Virtual reality (VR) provides an innovative approach for overcoming treatment barriers and may help address psychological risk factors for persistent pain and disability. However, the neural mechanisms underlying VR, particularly in subacute orthopedic pain, are insufficiently understood.

**Objective:**

This pilot study examined the feasibility, acceptability, and neural correlates of an 8-week home-based therapeutic VR intervention (RelieVRx) for subacute (<2 mo postinjury) musculoskeletal pain, using functional near-infrared spectroscopy (fNIRS) to assess changes in prefrontal cortex (PFC) activation and coactivation.

**Methods:**

Ten orthopedic patients (mean age 46.8, SD 11.86 years; 7/10, 70% female) completed the RelieVRx intervention and underwent fNIRS and behavioral assessments before and post treatment. Behavioral measures included pain intensity, pain interference, pain catastrophizing, pain anxiety, mindfulness, coping, and pain self-efficacy. fNIRS recorded PFC hemodynamic responses during movement-evoked pain and VR-based relaxation/distraction tasks. Feasibility and acceptability were assessed qualitatively and quantitatively (valid fNIRS recordings, participant feedback). Analyses evaluated pre-post changes in PFC activation, functional coactivation, and correlations with behavioral measures.

**Results:**

fNIRS procedures demonstrated high feasibility (74/80, 93% valid recordings), acceptability, and there were no safety concerns. Significant improvements were observed across all behavioral measures, including reduced pain intensity at rest (mean difference [MD]=−2.50, *P*<.001, *d*=2.24), and with activity (MD=–3.40, *P*<.001, *d*=1.98), decreased pain interference (MD range −3.90 to −4.90, *P*<.001, *d* range 1.32 to 2.30), reduced pain anxiety (MD=–32.70, *P*=.001, *d*=2.14) and pain catastrophizing (MD=–16.40, *P*=.003, *d*=2.13), and improved mindfulness (MD=+3.29, *P*=.01, *d*=0.94), coping (MD=+0.59, *P*=.01, *d*=1.01), and self-efficacy (MD=15.40, *P*=.008, *d*=1.51). fNIRS showed significant posttreatment increases in medial PFC activation (right medial channel: distraction task *t*=−4.473, *P*=.004; relaxation task *t*=–3.036, *P*=0.02) and enhanced coactivation between medial and lateral PFC regions (distraction task *t*=−2.784, *P*=.03). Increased functional coactivation between the right medial and left lateral PFC was negatively correlated with improved mindfulness (*r*=−0.716, *P*=.046) and coping scores (*r*=−0.709, *P*=.049).

**Conclusions:**

This study provides initial evidence of the feasibility and acceptability of integrating fNIRS neuroimaging into clinical VR interventions. Results indicate that engagement with VR therapy was associated with alterations in neural activity in key PFC regions implicated in pain regulation, correlating with significant improvements in pain and behavioral measures. The neural and behavioral changes highlight the potential of VR as a mechanistically informed, scalable nonpharmacological approach to managing subacute pain following orthopedic injuries. These findings justify larger trials that incorporate multimodal neuroimaging to further understand potential mechanistic processes that may underlie VR-based pain therapies.

## Introduction

### Gaps in Pain Management After Injury

Acute traumatic musculoskeletal injuries, such as fractures and dislocations, are both prevalent and costly [1]. Current standard of care for patients with musculoskeletal injuries primarily focuses on the physical aspects of recovery, including the immediate stabilization of injury (eg, surgery, casting, splinting), pharmacological pain management (eg, opioid and nonopioid analgesics), and physiotherapy. However, an estimated 20% to 50% of these cases result in persistent pain and functional limitations, even after the bones and soft tissues have healed [1-3]. These patients present a significant public health challenge and contribute substantially to health care costs and utilization due to the need for frequent medical appointments and multiple surgeries. Further, these injuries and subsequent pain have contributed to the United States opioid epidemic [4,5]. Poor recovery outcomes may be explained by psychological factors after injury, including pain catastrophizing (pain misconceptions, rumination), pain anxiety (fear or worry about pain), and pain self-efficacy (perceived ability to carry out daily activities despite pain), which increase the risk for chronic pain and disability independent of injury type, location, or severity [6]. Behavioral interventions, such as relaxation and mindfulness-based approaches, have been associated with enhanced coping and reduced pain intensity and interference among chronic populations [7,8], including orthopedic injuries [9,10]. However, access to behavioral interventions is often limited in orthopedic settings because treatments prioritize the physical aspects of recovery and due to barriers including mental health stigma, time, cost, transportation, and clinician availability [11,12].

Virtual reality (VR) may help overcome these treatment barriers and demonstrates growing evidence as a pain management tool [13,14]. VR is “a computer-generated simulation of the real or imagined environment or world” [15] that involves immersive, multisensory feedback and user interaction, most commonly delivered through a headset [16]. Immersion and interaction with multisensory VR stimuli, such as nature landscapes or games, is hypothesized to engage the user’s limited attentional resources, potentially diverting focus away from pain perceptions and related stress [14,17]. Most VR studies have focused on distraction to improve pain tolerance limits [17] and pain intensity [14] in chronic pain [18] or other acute pain populations (eg, burns and painful medical procedures) [14]. Additionally, VR provides a novel opportunity to target maladaptive pain responses by teaching relaxation and mindfulness skills. VR relaxation mindfulness studies report demonstrated high adherence and reductions in pain and pain catastrophizing in chronic pain populations [19-21] and feasibility in subacute orthopedic pain [22]. The mechanisms underlying VR distraction and relaxation remain poorly understood [17], particularly in subacute musculoskeletal pain, limiting our ability to identify the neural and psychological processes through which VR reduces pain.

### Role of the Prefrontal Cortex in Pain Modulation

The prefrontal cortex (PFC) plays a pivotal role in the modulation of pain through cognitive and emotional processes and includes various subregions such as the anterior PFC or frontopolar cortex (Brodmann area 10) [23], dorsolateral PFC (dlPFC) [24], ventromedial PFC [25], and orbitofrontal cortex [26]. These areas are integral to processes including attention, emotion regulation, and decision-making, all of which influence pain perception [27]. Neuroimaging studies have consistently shown that many of these PFC subregions are actively engaged in nociceptive processing, playing key roles in both pain perception and modulation [28]. Specifically, the anterior PFC or frontopolar cortex, encompassing medial and lateral Brodmann areas 10, extending into regions of Brodmann areas 12 and 32, is implicated in emotion regulation, decision-making, and cognitive appraisal of pain [29]. Neuroimaging studies have shown that medial PFC (mPFC) activity tracks expectations about pain and mediates expectancy effects on pain-related activity in other brain regions [30]. Functional near-infrared spectroscopy (fNIRS) studies, including our own, have reported robust activity in medial and lateral Brodmann area 10 during acute pain [31,32] that is suppressed by opioids [33,34]. Research investigating the neural correlates of stressor controllability in humans indicates that the mPFC mediates perceptions of control and subsequently regulates stress responses [35]. These findings suggest that mPFC, along with other subregions, could be investigated as potential neural targets for pain management interventions such as VR-based therapies [36].

Indeed, several studies have shown that brain regions engaged during VR overlap extensively with those implicated in chronic pain and behavioral interventions, including the dlPFC [37,38]. The dlPFC is a complex structure (spanning Brodmann areas 9, 8a, 8b, and 46) responsible for executive control, including the maintenance and regulation of top-down modulation and driving appropriate behavioral responses [39]. When compared with controls, increased dlPFC activation has been observed in response to painful stimuli in patients with chronic pain, such as fibromyalgia and knee osteoarthritis [40], likely due to central sensitization [41]. Activation of the dlPFC is associated with cognitive control of pain and decreased pain catastrophizing, suggesting its important role in pain coping [42]. These findings highlight the pivotal role of multiple PFC subregions in integrating cognitive, emotional, and evaluative processes to modulate pain, underscoring their importance as potential neural targets for pain management interventions such as VR-based therapies [36].

### fNIRS for VR Neuroimaging

fNIRS has gained significant attention for characterizing neural mechanisms of VR [43-45]. fNIRS uses low-energy near-infrared light to quantify cortical hemodynamic variations by measuring changes in oxygenated hemoglobin (HbO) and deoxygenated hemoglobin concentrations. Unlike other neuroimaging methods, fNIRS is generally less affected by electrical interference, making it highly compatible with VR headsets [43]. Additional advantages of fNIRS for VR research include that it (1) is noninvasive; (2) is relatively low-cost; (3) offers higher motion tolerance, enabling brain measurements during movement; (4) operates silently, which preserves the sense of immersion in VR environments [43,46]; and (5) is commonly used as a reliable measure of PFC activation and connectivity in pain [47,48]. Despite these advantages, only 2 studies have used fNIRS to characterize pain and VR-induced changes in PFC activity. Deng et al [49] used VR to divert participants’ attention while electrical pain stimuli were applied, resulting in significant activations in the dLPFC and premotor cortex. Hu et al [50] explored the brain mechanisms of mindful breathing using immersive VR and found that meditation increased pain thresholds, likely by enhancing PFC connectivity. These findings underscore the potential of fNIRS to capture dynamic changes in PFC activity related to pain and its modulation, providing a robust framework for investigating how different VR-based pain management strategies influence neural mechanisms in subacute pain after musculoskeletal injury.

### Study Aims and Hypotheses

The principal objective of this study was to explore the role of VR on neural mechanisms and behavioral measures for subacute pain following a pilot sample of acute traumatic musculoskeletal injury. We analyzed fNIRS data collected during a feasibility pilot study of an 8-week home-based therapeutic VR (RelieVRx) in 10 orthopedic patients with subacute pain after injury [22]. RelieVRx is Food and Drug Administration–authorized VR therapy for chronic lower back pain [51-53] and has demonstrated high feasibility, satisfaction, and preliminary improvements in orthopedic pain [22]. RelieVRx is a multicomponent therapeutic VR that includes both relaxation (eg, guided deep breathing) and distraction (eg, focused attention games) conditions, which enabled us to examine differential PFC activation patterns. We hypothesized that VR engagement would be associated with altered PFC hemodynamic activity and functional coactivation (FC), which refers to the temporal correlation between spatially distinct brain regions reflecting synchronized neural activity. Specifically, we expected that engagement in VR-based relaxation and distraction would lead to distinct patterns of PFC activation, with potential differences in medial versus lateral PFC engagement. Furthermore, we hypothesized that these neural adaptations would be associated with pre- and post-VR changes in behavioral measures, including pain outcomes (pain interference and intensity) and psychological mechanisms of action (pain catastrophizing, pain anxiety, pain self-efficacy, mindfulness, and coping).

We proposed 3 aims to understand the neural mechanisms and behavioral correlates of VR-based pain modulation in patients with subacute musculoskeletal injuries. First, we aimed to evaluate the feasibility of our fNIRS data collection procedures and the acceptability of fNIRS from patients’ perspectives using 30-minute qualitative exit interviews. This initial step to confirm the integrity of our data collection procedures and potential burden on patients is essential prior to subsequent mechanistic investigation because no study has used fNIRS to understand VR treatment effects after acute traumatic musculoskeletal injuries. Second, we investigated alterations in PFC activation and FC during 2 movement-evoked pain (injured and noninjured) and 2 VR (distraction and relaxation) conditions before and after participants completed the VR. Finally, we explored possible brain–behavior associations between pre- and post-VR changes in PFC activity and behavioral measures (pain outcomes, psychological mechanisms of action). The study aimed to provide a proof of concept for integrating VR and fNIRS neuroimaging in orthopedic populations. Consistent with the National Institute of Health Stage Model and National Center for Complementary and Integrative Health Research Framework, these aims establish a stepwise approach—from feasibility, to characterizing neural response, to brain-behavior relationships—designed to inform a subsequent mechanistic clinical trial of RelieVRx targeting reductions in pain interference after orthopedic injury.

## Methods

### Ethical Considerations

The study was approved by the Mass General Brigham Institutional Review Board (Protocol #2022P001500) and preregistered at ClinicalTrials.gov (NCT05552430). This study adheres to the CONSORT-EHEALTH (Consolidated Standards of Reporting Trials of Electronic and Mobile Health Applications and Online Telehealth) checklist [54]. The ancillary reviews by the Partners Research Information Security Office approved the VR (AppliedVR RelieVRx), and the Laser Safety Biomedical Engineering Review approved the fNIRS device (OctaMon, Artinis Medical Systems, The Netherlands). All participants provided written informed consent. The study protected participant privacy through secure data storage, restricted team access, encrypted transfers, coded identifiers, MGB-compliant communication, and use of a VR headset (Pico G2 4K) specifically selected to avoid the collection of personal information. Participants were compensated US $50 total (US $25 per assessment visit). Full methodological details, derived from a pilot feasibility study of RelieVRx for acute orthopedic injuries, are described in Mace et al [22]. Analyses of the fNIRS data have not been reported elsewhere.

### Participants

Participants with acute orthopedic traumatic musculoskeletal injuries were recruited through surgeon referrals at 2 Mass General Brigham Level 1 Trauma Clinics, as well as via flyers and the Partners Rally online research platform. Our target sample size of 10 participants was determined based on guidelines for pilots [55,56], National Institute of Health recommendations for testing feasibility [57,58], and similar pilot studies involving novel applications of fNIRS [38] and VR [18] for pain. While the sample size was appropriate for testing the feasibility of integrating fNIRS with VR and detecting neural responses [59-64] by design, it was not statistically powered for efficacy analysis or reproducibility.

Inclusion criteria were as follows: (1) age ≥18 years; (2) fluency and literacy in English; (3) upper or lower extremity orthopedic injury (eg, fracture, dislocation, or rupture) or surgical repair within the previous 2 months; (4) psychological risk for persistent pain and disability, indicated by a Pain Anxiety Symptom Scale-20 score ≥40 or a Pain Catastrophizing Scale-13 (PCS-13) score ≥20 [65,66]; (5) internet access; (6) willingness to participate and comply with the study protocol; (7) stable psychotropic medication regimen for >6 weeks; and (8) clearance by the referring orthopedic surgeon.

Exclusion criteria were as follows: (1) history of persistent pain (≥3 mo) prior to the injury; (2) complex polytrauma (≥1 injury location); (3) epilepsy, seizure disorder, dementia, migraines, or other neurological conditions; (4) nausea or dizziness; (5) light hypersensitivity; (6) significant vision or hearing impairment; (7) injuries to the eyes, face, or neck that hinder VR use; (8) medical conditions expected to worsen within 3 months; (9) untreated severe mental illness (eg, bipolar disorder, schizophrenia, active substance use); (10) surgical complications (eg, infection and need for repeat surgery); (11) ongoing litigation or Worker’s Comp claims; (12) pregnancy; (13) regular practice of cognitive-behavioral therapy or other mind-body techniques (>1/wk, ≥45 min).

As depicted in Multimedia Appendix 1, a total of 109 patients were referred for the study. Of these, 49 underwent screening, and 12 were deemed eligible. The primary reasons for exclusion were lack of clinically significant pain catastrophizing or pain anxiety (n=21), injury occurred more than 2 months ago (n=7), and history of chronic pain (n=6). Two eligible participants withdrew before the baseline visit. Table 1 presents the characteristics of the 10 participants who completed the VR and assessments. Multimedia Appendix 2 presents injury and treatment details for all participants at baseline. All participants were right-handed.

**Table 1.** Demographics and clinical characteristics (n=10).

Characteristics	Values
Age (years), mean (SD)	46.8 (11.86)
Time since injury (days), mean (SD)	38.40 (36.33)
Time since surgery (days), mean (SD)	23.29 (17.32)
Engagement in physiotherapy (d/w), mean (SD)	2.83 (2.14)
Gender, n (%)	
Woman	7 (70.0)
Man	3 (30.0)
Ethnicity, n (%)	
Not Hispanic or Latino/Latina	10 (100.0)
Race, n (%)	
White	10 (100.0)
Marital status, n (%)	
Married	3 (30.0)
Single, never married	5 (50.0)
Separated or Divorced	1 (10.0)
Widowed	1 (10.0)
Education, n (%)	
Some college/Associates degree (<16 y)	1 (10.0)
Completed college (16 y)	3 (30.0)
Graduate/professional degree (>16 y)	6 (60.0)
Employment, n (%)	
Employed full-time	8 (80.0)
Employed part-time	1 (10.0)
Unemployed	1 (10.0)
Income (US $), n (%)	
15,000 to less than 20,000	1 (10.0)
50,000 to less than 75,000	3 (30.0)
75,000 or more	6 (60.0)
Injury location, n (%)	
Tibia	1 (10.0)
Ankle	2 (20.0)
Foot	1 (10.0)
Humerus	1 (10.0)
Wrist	2 (20.0)
Finger	3 (30.0)
Injury type, n (%)	
Fracture	9 (90.0)
Rupture	1 (10.0)
Surgery, n (%)	
Yes	7 (70.0)
No	3 (30.0)
Physiotherapy, n (%)	
Yes	6 (60.0)
No	4 (40.0)
Past year prior pain, n (%)	
Yes	3 (30.0)
No	7 (70.0)
Depression diagnosis, n (%)	
Previously	1 (10.0)
Currently	3 (30.0)
Never diagnosed	6 (60.0)
Anxiety, n (%)	
Currently	3 (30.0)
Never diagnosed	7 (70.0)
Posttraumatic stress disorder (PTSD), n (%)	
Currently	1 (10.0)
Never diagnosed	9 (90.0)
Substance use, n (%)	
Never diagnosed	10 (100.0)
Psychotropic medications, n (%)	
Yes	3 (30.0)
No	7 (70.0)
Nonnarcotic medications, n (%)	
Yes	6 (60.0)
No	4 (40.0)

### Enrollment Procedures

Procedures were based on prior orthopedic behavioral intervention trials [61,67] and VR pilot studies [51,52,68]. A research assistant screened participants for eligibility and scheduled baseline, pre-VR, and post-VR study visits at our research center. At the baseline visit, participants provided informed consent, completed assessments including fNIRS, and received a VR demonstration. The VR came with a travel case, charger, cleaning cloth, and user instructions. After the 8-week VR intervention, participants repeated the assessments and returned the VR headset.

### VR Intervention

Participants received a rental Pico G2 4K headset preloaded with the therapeutic VR (RelieVRx). The Pico G2 4K headset is lightweight (278 g), high-resolution (4K VR 3840 × 2160 display, 75 Hz refresh rate), and affordable ($245). The headset has several user-friendly features, including minimal setup, compatibility with glasses, and “hands-free” controls via the user’s head movements. The RelieVRx intervention, developed by AppliedVR [51], is a self-guided therapeutic program based on the biopsychosocial model of pain, incorporating cognitive behavioral therapy and mindfulness principles. The primary treatment components are VR relaxation and distraction. Relaxation conditions guide the participant through progressive relaxation exercises that are enhanced by biofeedback and immersive environments (eg, deep breathing while watching a sunset and observing the breath). Distraction conditions involve interactive focused attention games (eg, earning points by tracking butterflies amid distractor visual stimuli). Participants also receive pain neuroscience education to explain the biopsychosocial aspects of pain and the role of relaxation and distraction techniques (eg, visualizing pain reduction through nervous system calming). Over 8 weeks, participants completed one daily module (total=56 conditions, average=6 min, range 2 to 16 min) in a fixed sequence. The conditions are designed to be accessible and minimize potential risks (eg, emotional distress or motion sickness).

### Measures

#### Behavioral Measures

To explore brain–behavior relationships, we included several measures of pain outcomes (pain intensity and interference) and psychological mechanisms of action targeted by VR (pain catastrophizing, pain anxiety, pain self-efficacy, mindfulness, and coping). Pain intensity at rest and with activity in the last week was measured using the 2-item (0=“no pain”; 10=“worst ever”) Numerical Rating Scale. The Numerical Rating Scale is reliable and valid in orthopedic populations [69]. Pain interference with activity, sleep, mood, and stress was measured with the 4-item (0=“does not interfere”; 10=“completely interferes”) Defense and Veterans Pain Rating Scale [70]. The Defense and Veterans Pain Rating Scale was the primary outcome of RelieVRx trials [51,53,68] and is reliable and valid in acute and persistent pain populations [71]. Pain catastrophizing was measured using the 13-item (0=“not at all”; 4=“all the time”) PCS [72]. The PCS-13 (range 0‐52) is reliable and valid in populations with musculoskeletal pain [73]. Fear and anxiety related to pain were measured using the 20-item (0=“never”; 5=“always”) Pain Anxiety Symptoms Scale [74]. The Pain Anxiety Symptoms Scale-20 (range 0‐100) is reliable and valid in pain populations [75]. Pain self-efficacy was measured using the 10-item (0=“not at all confident”; 6=“completely confident”) Pain Self-Efficacy Questionnaire [76]. The Pain Self-Efficacy Questionnaire (range 0‐60) is reliable and valid in chronic pain populations [76]. Mindfulness was measured using the 12-item (1=“rarely/not at all”; 4=“almost always”) Cognitive and Affective Mindfulness Scale – Revised [77]. The Cognitive and Affective Mindfulness Scale – Revised (range 12‐48) has been used to measure mindfulness in pain [60,78], including orthopedic populations [79]. Coping was measured (0=“I cannot do this at all”; 4=“I can do this extremely well”) using the 13-item Measure of Current Status [80]. The Measure of Current Status (range 0‐52) is a reliable and valid measure of healthy coping skills in pain [60,78] and orthopedic populations [79].

#### fNIRS

##### Overview

**Figure 1.**
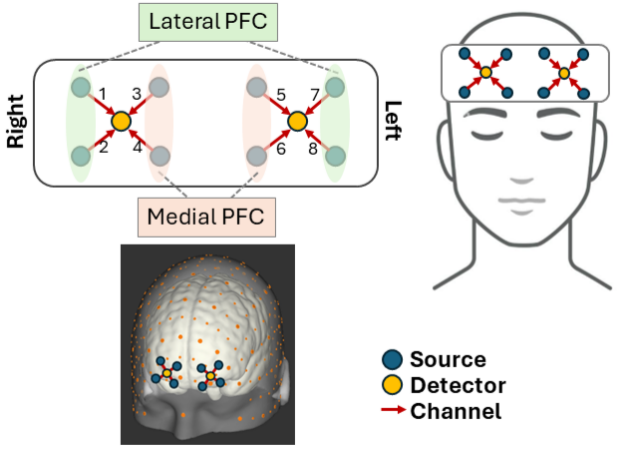
Artinis OctaMon 8-channel optical topography functional near-infrared spectroscopy (fNIRS) system. PFC: prefrontal cortex.

We measured changes in PFC activation in response to acute pain and VR with a wireless 8-channel fNIRS optical topography system (OctaMon, Artinis Medical Systems, The Netherlands). The PFC is divided into 4 regions in each hemisphere: 2 medial and 2 lateral (Figure 1 displays sensor layout details). fNIRS measures changes in HbO and deoxygenated hemoglobin concentrations through the propagation of near-infrared light between LED emitters and receivers [17]. The OctaMon system used in this study uses 2 wavelengths of light at 760 nm and 850 nm [81]. This approach shares a similar physiological basis as the blood-oxygenation-level-dependent signal activation in traditional functional magnetic resonance imaging.

##### fNIRS Data Acquisition

Participants underwent OctaMon fNIRS recordings after completing the self-reports during the baseline and postintervention visits. Participants sat in a comfortable stationary chair. The trained research assistant and principal investigator placed the standard OctaMon head cap on the participant’s forehead and firmly attached it with the side straps following standard operating procedures. The head cap had 8 mounted emitters (4 on each hemisphere) and 2 detectors (1 on each hemisphere) arranged in the OctaMon standard octode template over the PFC. The signal sampling rate was 10 Hz using a 2×4 emitter-detector channel layout. The differential pathlength factor was calculated based on participant age (range 6.00‐6.61 years). Participants were asked about their comfort level wearing the fNIRS, and none reported any problems. Additionally, the principal investigator (RAM) and research assistant monitored the continuous fNIRS data during the experiments to reduce potential confounds (eg, movement or ambient light) to ensure data quality. The headset was adjusted to improve signal quality as needed (eg, moving hair underneath optodes, ensuring skin contact). Raw fNIRS data were collected in Oxysoft (Artinis Medical Systems, The Netherlands).

##### fNIRS Experimental Design

###### Overview

**Figure 2.**
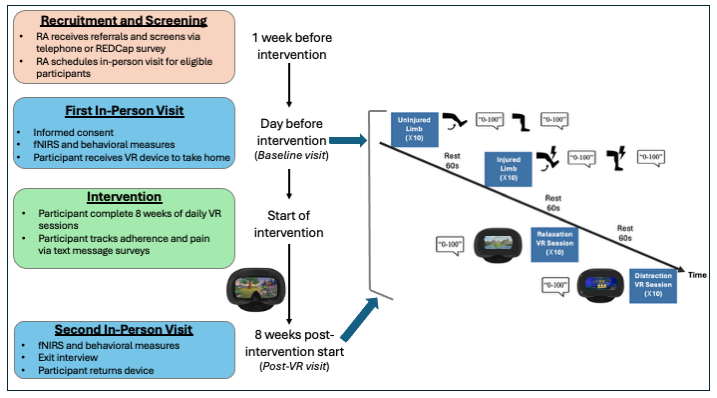
An overview of the experimental design. fNIRS: functional near-infrared spectroscopy; RA: research assistant; REDCap: Research Electronic Data Capture; VR: virtual reality.

An overview of the experimental design is presented in Figure 2. We describe the movement-evoked pain (injured and noninjured) and VR (distraction and relaxation) conditions in detail below.

###### Movement-Evoked Pain Conditions

We instituted a standardized movement-evoked pain paradigm across participants and sessions to maximize internal consistency while preserving ecological validity. First, a scripted instruction set was delivered verbatim at every visit to avoid variation in verbal cues. The tasks began from a fixed starting posture and joint-specific neutral alignment, determined with orthopedic surgeon collaborators, and participants maintained consistent body posture throughout. A predefined plane of movement was specified for each injured joint (eg, sagittal flexion/extension for wrist or knee), and participants performed active movement only (no external assistance) at a slow, uniform tempo (≈2‐3 s to end range) into the first pain threshold, held that position for 5 seconds, then relaxed for 3 seconds. After the initial hold, participants were instructed to move approximately 5 degrees further (visually estimated from anatomical landmarks) and hold for another 5 seconds, followed by return to rest. This hold–move–hold cycle was repeated 10 times per side with immediate pain ratings at the end of each hold (0‐100 for granular repeated pain ratings), both to confirm pain evocation and to improve fNIRS block reliability (ie, signal averaging) [82].

Participants always performed the noninjured (control) side first, which provided a within-participant reference of minimal pain movement, followed by the injured side for movement-provoked pain. This ordering is consistent with orthopedic examination workflows and supports external validity for real-world limb testing. To standardize the “five degrees more” increment, examiners used trained visual estimation anchored to anatomical landmarks (eg, joint lines and bony prominences) and practiced on exemplar trials prior to data collection. Although visual estimation is known to introduce measurement error relative to devices, it is common in clinical range of motion exams (eg, in gait or outpatient orthopedic settings) and has acceptable clinical utility [83]. Across all visits, movement speed, plane, posture, and tempo remained constant. The same scripted instructions, starting alignment, and tempo were preserved from session to session. We marked the start and end of each block on the time series fNIRS data using OxySoft event labels. We reset the graph traces before and after we marked each block to reorient the device for each condition. Participants were given 2 minutes to rest before the VR conditions.

###### Relaxation and Distraction VR Conditions

Participants completed two 6-minute VR conditions while wearing the fNIRS. We designed 2 conditions to measure PFC activation associated with 2 mechanisms of VR pain modulation: relaxation and distraction. Relaxation is intended to downregulate the sympathetic nervous system response to pain and related stress [17,84], while distraction aims to divert attention away from pain and stress signaling [85,86]. The order of the tasks was fixed to preserve the standardized sequence of the RelieVRx program and to maintain data quality during fNIRS acquisition. We considered counterbalancing, but it was not feasible without removing the fNIRS equipment, which would have disrupted signal quality and intervention fidelity. The relaxation condition was a 6-minute guided relaxation in a calming outdoor environment (module 6 of RelieVRx). Participants were instructed to notice internal physical sensations while showing and deepening their breath. Using the same range as the movement-evoked pain condition, participants rated their overall relaxation on a scale of 0 (“Not relaxed at all”) to 100 (“Very relaxed”) as an experimental manipulation check. The distraction condition was a 6-minute concentration game (module 8 of RelieVRx). Participants followed objects with their gaze to earn points across 3 levels of increasing difficulty: (1) tracking a single butterfly, (2) tracking a butterfly with a target color, and (3) tracking a butterfly with a target color among foil butterflies. Participants rated their overall focus on the visual target stimuli on a scale of 0 (“Not focused at all”) to 100 (“Very focused”) as a proxy for distraction from pain. To increase immersion, overhead lights were dimmed, participants wore headphones connected to the headset, and the study staff left the room during both VR conditions.

### Individual-Level fNIRS Data Analyses

#### fNIRS Data Preprocessing

fNIRS data preprocessing was performed with a combination of OxySoft, Homer2 [87], and customized scripts written in MATLAB (Mathworks). Preprocessing included the following steps. First, initial quality checks were performed using OxySoft [88], a proprietary Artinis Medical Systems software platform that provides a live signal display feature for monitoring head motion artifacts. Large and abrupt changes in signal intensity, indicative of excessive head motion, were manually marked and trimmed in real-time to mitigate motion-induced noise and maintain high fidelity in fNIRS recordings. For fNIRS data that passed the quality check, raw light intensity data were then converted into optical density change using hmrIntensity2OD function in Homer2 [87]. We automatically flagged brief motion-related spikes on each channel using a standard algorithm from the Homer2 toolbox that marks samples when the instantaneous jump in the light signal exceeds an amplitude threshold or when the short-term variability exceeds an SD threshold. We used a 0.5 s window with a 1 s mask, a 5-SD criterion for variability, and an amplitude criterion of 0.1 in optical-density units. Motion-correlated optical density data were further filtered with a third-order Butterworth bandpass filter, a commonly used filtering approach in fNIRS signal processing to remove high-frequency noise, such as cardiac and respiratory artifacts, and low-frequency drifts [89]. with high and low cutoff frequencies of 0.2 Hz and 0.01 Hz, respectively. After artifact screening and band-pass filtering (0.01‐0.20 Hz), we converted optical-density data to oxy- and deoxy-hemoglobin concentration changes using the modified Beer–Lambert law with age-adjusted differential pathlength factors. In this study, we focused on the analysis of the HbO time courses as HbO changes are reported to have a much higher signal-to-noise ratio and may have greater sensitivity to task-evoked changes [90].

#### Computation of Brain Imaging and Effect Size Metrics

Three types of brain imaging measures were then calculated based on the preprocessed fNIRS signals.

##### Task-Induced Activation Using General Linear Modeling

The neural activation magnitude for each region of interest was measured using a general linear model approach, which modeled the relationship between the hemodynamic response and task conditions by convolving the timing of the tasks with a canonical hemodynamic response function to estimate beta coefficients representing activation strength [91].

##### Task-Induced Change in HbO Using Effect Size

Additionally, for each condition (injured, noninjured, VR distraction, VR relaxation), we calculated an effect size metric (Cohen *d*) by taking the difference between the mean signal during the condition and the mean signal during the prestimulus baseline interval [38].

##### State-Related Functional Activity During VR

Pair-wise FC was computed using the correlation of HbO concentration time series among PFC subregions. FC measures can help understand how different regions of the PFC are engaged as a network during a given brain state (eg, pain or VR). Pearson correlation coefficients were calculated for all pair-wise combinations of channels within and across hemispheres to assess FC [92]. We further derived cross-regional metrics by averaging correlation values between specific channel pairs (eg, medial-left PFC and lateral-right PFC).

### Statistical Analyses

For Aim 1, the feasibility and acceptability of fNIRS data collection were assessed using a mixed methods approach. Descriptive statistics (eg, completion rates and signal quality metrics) were used to evaluate the feasibility of fNIRS data collection. Reasons for missing or invalid fNIRS recordings were documented and categorized to provide further insight into procedural challenges. We conducted a 30-minute in-person or remote (participant preference) individual exit interview during the postintervention visit to further understand participants’ perceptions of the fNIRS data collection. We used rapid assessment procedures to analyze the transcripts consistent with established qualitative frameworks [93-95]. Rapid assessment is a valid alternative to in-depth qualitative methods for generating timely, actionable insights to guide [96,97]. All authors met to collaboratively discuss each exit interview, discuss key content, and summarize emergent themes in a shared matrix to identify patterns across participants [98]. All authors collaboratively reviewed each transcript, discussed key content, and summarized emergent themes in a shared matrix to identify patterns across participants. Two team members independently completed the rapid assessments, and discrepancies were adjudicated by the principal investigator (RAM).

For Aim 2, paired sample *t* tests were conducted to compare baseline and posttreatment fNIRS measures for each participant, focusing on regional activation magnitudes and pair-wise FC across 4 conditions: movement-evoked pain in the injured and noninjured extremities and VR distraction and relaxation. These analyses aimed to identify significant neural changes following VR therapy. To further localize statistically significant hemodynamic activations at the group level, paired sample *t* tests were applied to the Cohen *d* parameter for each fNIRS channel separately. Cohen *d* was computed by taking the difference between the mean signal in the time range of the condition and the mean signal in the prestimulus baseline interval. This approach was adapted from previous fNIRS studies using Cohen *d* as a standardized effect size metric for assessing neural activation changes [38]. For each participant, Cohen *d* values were calculated for movement-evoked pain and VR conditions at both baseline and posttreatment. Multiple comparison corrections were applied using the false discovery rate [99].

For Aim 3, Pearson’s correlation analyses were performed to examine associations between pre-to-post VR changes in PFC activity and FC and changes in behavioral outcomes. Specifically, correlations were conducted between statistically significant neural response changes and self-reported pain outcomes (pain interference, pain intensity) and psychological mechanisms of action (pain catastrophizing, pain anxiety, pain self-efficacy, mindfulness, and coping).

Finally, to assess potential effects of sample heterogeneity, we examined associations between time since injury and (1) baseline measures and (2) change scores (postbaseline) using Spearman correlations with Benjamini–Hochberg correction. We performed sensitivity analyses excluding the single 138-day case and using leave-one-out and robust regression. We further modeled postintervention outcomes via analysis of covariance, including baseline value, time since injury, and injury type (collapsed categories) as covariates. Injury type and time since injury were not significant predictors, and model inferences for the intervention remain unchanged.

## Results

### Aim 1: Feasibility and Acceptability

Of the 80 possible fNIRS recordings (ie, 10 participants, 2 time points, 4 conditions [pain, no pain, distraction, and relaxation]), 74 (93%) of them resulted in valid data that were included in the analysis, suggesting high feasibility of fNIRS data collection. Five recordings were missing, and one recording was invalid due to optode signal interference. Manipulation checks provided internal validity that injured movements evoked more pain (mean 44.11, SD 17.17) than the noninjured movements (mean 2.74, SD 7.60). Similarly, manipulation checks indicated that the VR conditions elicited high relaxation ratings (mean 80.25, SD 11.19) and distraction ratings (mean 82.50, SD 11.40) as intended (0=min, 100=max). No major safety concerns related to VR or fNIRS were noted during the study. Participants reported no discomfort or VR sickness.

Exit interviews revealed that fNIRS was acceptable to participants. Participants described the device as noninvasive, comfortable, and easy to use. Prior to the study, none of the participants had prior knowledge of fNIRS. They found the informational pamphlet helpful before the initial visit. They valued the study team’s emphasis on safety and appreciated opportunities to ask questions. Several participants viewed fNIRS as more accessible than MRI. Advance notice regarding hair adjustments prevented discomfort. They requested more information about the purpose and interpretation of fNIRS data, and they affirmed the importance of institutional approvals (eg, their orthopedic surgeon’s involvement). Some indicated that it felt cumbersome to wear fNIRS and VR equipment. Despite these inconveniences, participants expressed willingness to use fNIRS in future orthopedic research and showed interest in the results.

### VR Therapy and Behavioral Measures

We observed significant and large pre-post VR reductions in all outcomes, including decreases in pain intensity at rest (mean difference [MD]=−2.50, *P*<.001, *d*=2.24), pain intensity with activity (MD=−3.40, *P*<.001, *d*=1.98), and pain interference (MD range −3.90 to −4.90, *P*<.001, *d* range 1.32 to 2.30). Similarly, we also found significant and large pre-post VR improvements in the mechanisms of action. Pain anxiety (MD=−32.70, *P*=.001, *d*=2.14) and pain catastrophizing (MD=−16.40, *P*=.003, *d*=2.13) significantly decreased, while pain self-efficacy (MD=+15.40, *P*=.008, *d*=1.51), mindfulness (MD=+3.29, *P*=.01, *d*=0.94), and coping (MD=+0.59, *P*=.01, *d*=1.01) significantly increased.

### Aim 2: Group-Level Hemodynamic Activation

Regarding hemodynamic activation magnitudes (ie, beta value measures) for the VR distraction and VR relaxation conditions at baseline and posttreatment, significant differences in activation were observed between pre and posttreatment fNIRS recordings. Prior to the initiation of the noninjured condition, a significant reduction in HbO concentration was observed at channel 8, corresponding to the left lateral PFC (*t*=−2.585, *P*=.03).

Analysis of changes in Cohen *d* parameter between baseline and posttreatment fNIRS measurements revealed a significant increase in HbO at channel 4, which represents the right mPFC. Specifically, this increase was significant for both conditions (VR distraction condition: *t*=−4.473, *P*=.004; VR relaxation: *t*=−3.036, *P*=.03).

### Aim 2: Functional Coactivation Analysis

**Figure 3.**
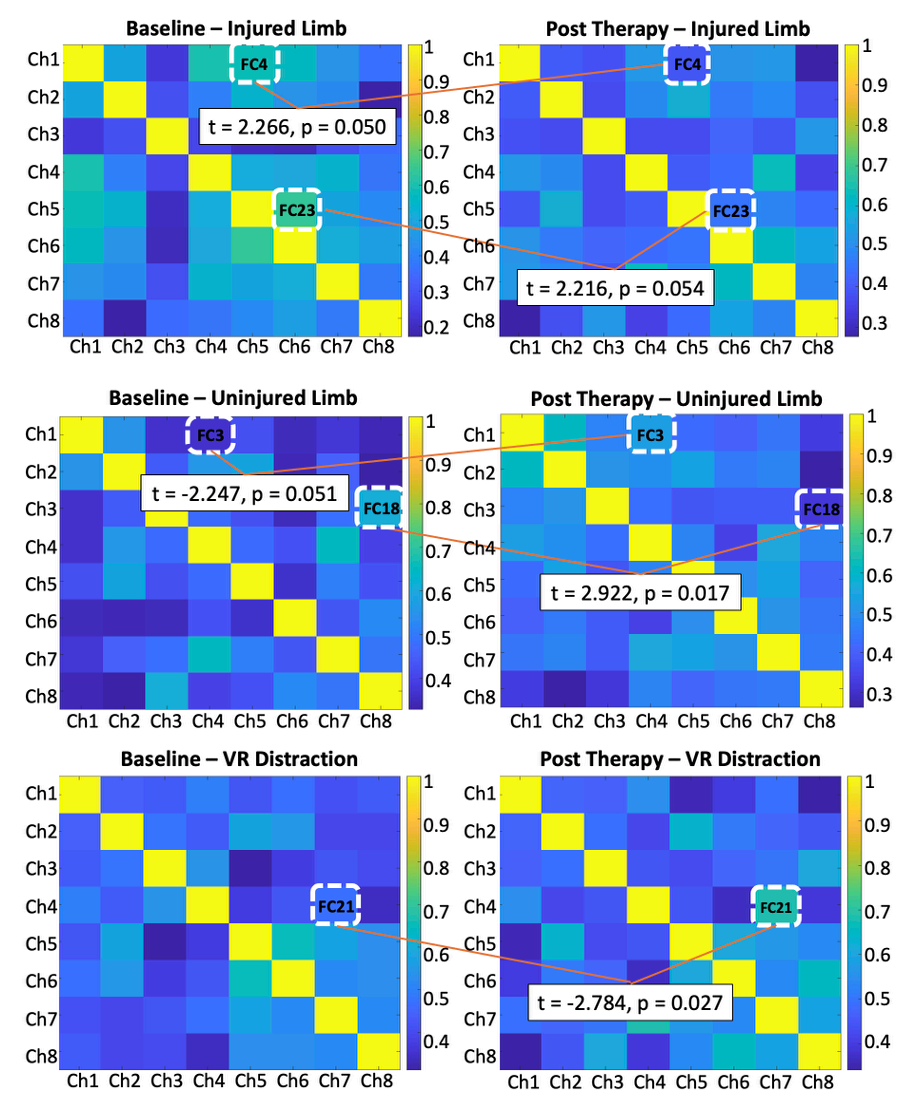
Distinct patterns of functional connectivity between baseline and posttreatment conditions. Ch: channel; FC: functional coactivation; *P*: statistical significance; *t*: average difference; VR: virtual reality.

FC analyses identified significant differences between baseline and posttreatment conditions across multiple comparisons (Figure 3). In the injured condition, changes were observed in FC23 (*t*=2.216, *P*=.05), representing coactivation within the left mPFC, and FC4 (*t*=2.266, *P*=.05), representing coactivation between the right lateral PFC and left mPFC. In the noninjured condition, alterations were detected in FC18 (*t*=2.922, *P*=.02), which represents coactivation between the right mPFC and left lateral PFC, and FC3 (*t*=–2.247, *P*=.05), representing coactivation between the right lateral PFC and right mPFC. In the VR distraction condition, significant differences were identified in FC21 (*t*=−2.784, *P*=.03), which represents coactivation between the right mPFC and left lateral PFC.

### Aim 3: Brain-Behavior Associations

**Figure 4.**
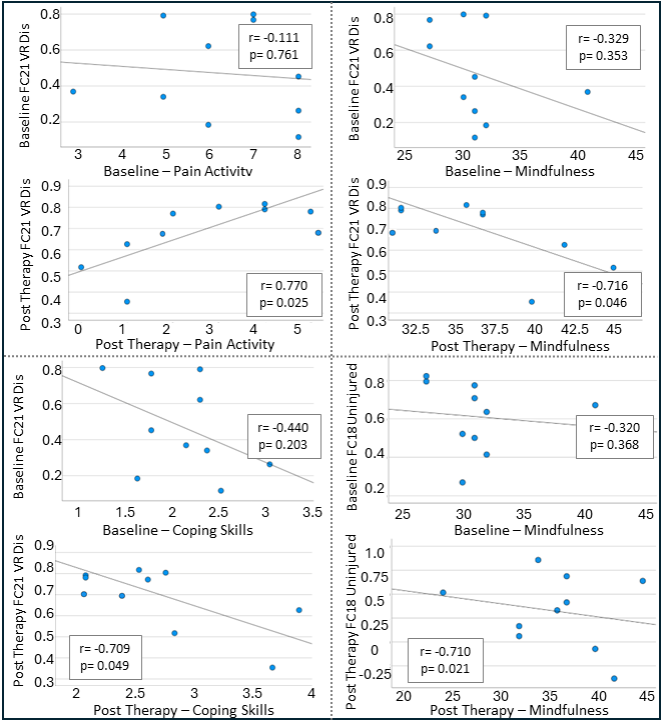
Brain-behavior correlation results. The x-axis refers to behavior measures of pain activity (Numerical Rating Scale [NRS]), mindfulness (Cognitive and Affective Mindfulness Scale – Revised, CAMS-R), and coping skills (measure of current status, MOCS) completed at the baseline and postintervention visits. FC: functional coactivation; VRDi: virtual reality distraction condition.

To investigate the relationship between HbO response changes and behavioral outcomes, correlations between alterations in Cohen *d* parameters and self-reported measures were examined. A significant correlation was observed between the hemodynamic response to painful stimuli at channel 4 (right mPFC) and pain-related activity, with greater increases in pain levels associated with increased activation in this region (*r*=0.760, *P*=.047). FC analyses further revealed key findings (Figure 4). In the VR distraction condition, baseline FC21 (right mPFC – left lateral PFC activity) was not significantly correlated with pain activity (*r*=−0.111, *P*=.76). However, posttreatment FC21 demonstrated a strong positive correlation with pain activity (*r*=0.770, *P*=.02). Additionally, posttreatment FC21 (right mPFC – left lateral PFC activity) exhibited significant negative correlations with mindfulness (*r*=−0.716, *P*=.046) and coping skills (*r*=−0.709, *P*=.049); however, such correlation patterns were not observed at baseline (mindfulness: *r*=−0.329, *P*=.35; coping skills: *r*=−0.440, *P*=.20). Additionally, in the noninjured condition, posttreatment FC18 (right mPFC – left lateral PFC activity) showed a significant negative correlation with mindfulness (*r*=−0.710, *P*=.02), whereas no significant correlation was observed at baseline (*r*=−0.320, *P*=.37).

## Discussion

### Principal Findings

This study systematically investigated the neural mechanisms and behavioral measures underlying pain modulation in patients with recent acute musculoskeletal injuries undergoing an 8-week home-based therapeutic VR intervention. fNIRS characterized PFC activity and FC in response to VR distraction and relaxation conditions, as well as movement-evoked pain. Our findings indicate significant neurophysiological adaptations in the PFC alongside significant and large improvements in patient-reported pain outcomes (interference and intensity) and psychological mechanisms (pain catastrophizing, pain anxiety, pain self-efficacy, mindfulness, and coping), reinforcing the potential of VR therapy as a clinically viable nonpharmacological treatment for subacute pain after musculoskeletal injury. Notably, this interventional study complements cross-sectional and provides preliminary evidence of pre-post differences in brain–behavior associations during a VR program. Our study contributes to the literature by suggesting (1) the feasibility of integrating fNIRS into clinical trial settings to evaluate nonpharmacological pain treatments and (2) the utility of combining neuroimaging and behavioral measures to improve understanding of treatment mechanisms and therapeutic effects. Given the small sample size and single-arm design, the observed associations between VR and neurobehavioral adaptations are preliminary and should be interpreted with caution, as described in the sections below.

### Differentiated Neural Response Patterns in the PFC

A primary objective of this study was to elucidate the role of the PFC in VR-mediated pain modulation. Our findings show alterations in hemodynamic activity and FC during painful movement across multiple PFC subregions after VR therapy, reinforcing the central role of PFC in top-down pain regulation [100] and its potential as a neurobiological target for VR-based pain therapy. The PFC plays a crucial role in pain perception, integrating cognitive, affective, and sensory components of pain processing, with the medial and dorsolateral areas of PFC particularly implicated in cognitive control mechanisms, including pain suppression and emotion regulation [27,39].

In chronic pain conditions, hyperactivity in the dlPFC is often associated with increased pain perception, reflecting its role in pain chronification rather than actively suppressing pain [37,101]. Conversely, effective behavioral pain management approaches, including cognitive behavioral therapy and mindfulness-based interventions, have been shown to normalize or reduce excessive PFC activity, suggesting an adaptive regulatory role [102,103]. Our findings indicate that VR therapy may similarly engage PFC mechanisms involved in cognitive pain modulation. Increased activation in the right mPFC (channel 4) following both VR distraction and relaxation may reflect increased engagement of neural processes implicated in pain regulation. Prior neuroimaging studies have linked the mPFC to cognitive reappraisal of pain, self-referential processing, and emotion regulation [104,105], indicating that VR therapy may strengthen these adaptive neural pathways. Increased activation in the mPFC during both conditions aligns with prior findings that distraction-based VR predominantly engages attentional networks, while relaxation-based VR enhances interoceptive awareness and downregulates stress responses [106,107]. These findings underscore the need to further delineate these overlapping but functionally distinct neural networks involved in VR-based pain management strategies.

Beyond regional activation, modulations in PFC coactivation helped characterize state-dependent network engagement across conditions. The presence of comparable or stronger connectivity changes during uninjured limb movements indicates that these effects are not specific to nociceptive input, but instead likely reflect domain-general regulatory and attentional processes that are engaged during movement and modulated by VR. Notably, FC21 (right mPFC–left lateral PFC coactivation during VR distraction) exhibited a significant increase following VR therapy. While postintervention associations between medial–lateral PFC coactivation and behavioral outcomes are consistent with models of pain regulation, the absence of corresponding baseline relationships precludes conclusions about stable individual differences in regulatory capacity. This pathway has been associated with executive control and the integration of affective and sensory aspects of pain processing [39]. However, rather than reflecting a monotonic marker of regulatory efficiency, medial–lateral PFC coactivation likely indexes regulatory demand or engagement, with greater coupling occurring when pain-related cognitive control requirements are higher. Under this interpretation, increased FC21 may reflect compensatory recruitment of control networks in the presence of greater pain or task demands, rather than direct evidence of improved regulation.

Although potentially counterintuitive, the inverse associations between medial–lateral PFC coactivation and mindfulness/coping are consistent with models in which effective self-regulation reflects reduced reliance on concurrent default-mode network (DMN) and executive-control network engagement, indicating greater functional segregation and more efficient network switching. In this framework, individuals with stronger psychological resources may require less medial–lateral PFC coactivation to perform the same task, whereas individuals experiencing greater pain or lower coping capacity may exhibit increased coactivation as a compensatory response. Thus, individuals with stronger psychological resources (such as mindfulness) may require less concurrent DMN–executive control networks engagement during the VR distraction task, yielding lower medial–lateral coactivation after treatment. Conversely, people with lower coping skills may require greater PFC engagement to perform the same task. This view is supported by DMN–control anti-correlations observed during adaptive regulation [36,100,108], by prior fNIRS findings of opposing mPFC versus lateral PFC responses under analgesic/pain-modulatory states [23], and by chronic-pain literature linking disrupted DMN connectivity (including DMN–mPFC coupling) to pain rumination [40,105]. Collectively, the present inverse correlations may reflect recovery/normalization of DMN–executive control networks interactions following VR training, with potential trait-dependent differential effects on attentional control and coping; however, these findings are exploratory and merit replication in larger mechanistic cohorts. These findings contribute to the growing body of evidence demonstrating that therapeutic interventions and their relationships with behavioral outcomes can dynamically modulate regional and network activity in the PFC.

### Therapeutic Implications

Analyses of brain–behavior correlates provided further evidence of the therapeutic potential of VR interventions for subacute pain management. A significant correlation was observed between the hemodynamic response to painful stimuli at channel 4 (right mPFC) and self-reported pain, such that greater pain intensity was associated with increased activation in this region during VR distraction. This finding implicates the mPFC in the affective processing of pain and suggests that interventions capable of dampening mPFC hyperactivity, such as VR distraction, may attenuate perceived pain intensity [104,105]. Additionally, mindfulness and coping were negatively associated with FC between the right medial and left lateral PFC (FC18 during uninjured limb movement, and FC21 during VR distraction) following treatment. These associations suggest that medial–lateral PFC coactivation reflects context-dependent regulatory engagement, such that individuals with stronger psychological resources may achieve regulation with reduced concurrent PFC coupling, whereas greater coactivation may reflect increased regulatory effort in the presence of higher pain or affective demand. Specifically, individuals with higher mindfulness may require less effortful top-down control to manage pain, consistent with evidence that mindfulness training reduces prefrontal over-engagement and promotes more automatic emotion regulation [108-110]. Although significant associations were observed between neural and psychological outcomes, the direction of causality remains uncertain. It is unclear whether changes in brain coactivation facilitated improvements in mindfulness and coping or if psychological gains induced neurophysiological adaptation. Nevertheless, these findings align with prior research emphasizing the critical role of cognitive-affective PFC networks in adaptive pain modulation [111] and underscore the potential of VR-based interventions to foster beneficial neurobehavioral adaptations.

Last, several nonexclusive explanations may account for why brain–behavior relationships were observed only postintervention. First, limited measurement reliability or restricted variance at baseline—particularly within a subacute pain cohort—may have attenuated detectable associations prior to intervention. Second, postintervention associations may be confounded by unmeasured factors such as engagement or expectancy that covary with both neural responses and self-reported outcomes. Third, given the modest sample size and multiple exploratory tests, posttreatment correlations have the risk of reflecting type I error.

Taken together, the emergence of postintervention brain–behavior correlations, though preliminary, suggests potentially meaningful VR-related neuroadaptations. For instance, the positive association between medial–lateral PFC coactivation and pain activity serves as a dynamic rather than a unidimensional marker of treatment success. It likely reflects a state-dependent coupling metric that varies as a function of pain intensity, cognitive demand, and regulatory capacity, potentially indexing compensatory engagement or regulatory effort. Though the single-arm design and modest sample size preclude a definitive causal mechanism, these findings provide an empirical foundation for evaluating VR-based pain interventions. By identifying specific neurobiological targets, these findings are intended to inform the design of future randomized, adequately powered mechanistic studies incorporating appropriate control conditions, repeated longitudinal neural assessments, and direct measurement of engagement-related factors to rigorously test how brain–behavior relationships evolve with intervention.

### Limitations

Five limitations warrant consideration: (1) fNIRS spatial resolution, (2) movement-evoked pain variability, (3) sample size, (4) single-arm design, and (5) fixed VR protocol.

First, the restricted spatial coverage of fNIRS limits insights into broader pain-processing networks, including the insula, anterior cingulate cortex, and sensorimotor regions—key areas in pain perception and modulation [24]. fNIRS lacks the spatial resolution to precisely localize activity within PFC, highlighting the need for complementary neuroimaging methods like functional magnetic resonance imaging or high-density fNIRS to refine our understanding of prefrontal involvement in VR-mediated pain processing.

Second, individual variability in pain and range of motion introduces heterogeneity in our movement-evoked pain condition, despite our protocol to standardize posture, plan, speed, and scripted instructions. These constraints reflect the ecological intent of the protocol; yet, future trials should use device-based angle capture (eg, digital inclinometers) to reduce variability and consider including additional standardized pain assessments (eg, quantitative sensory testing).

Third, the small sample size (N=10) constrains generalizability. However, this study was explicitly designed as a feasibility pilot to evaluate the acceptability and technical integration of fNIRS with a therapeutic VR intervention in subacute pain. In line with established guidance for pilot studies [18,38,55-58], findings from this sample are intended to be descriptive and hypothesis-generating rather than confirmatory. Small samples can produce unstable effect estimates and elevate both type I and type II error risk; accordingly, observed brain–behavior associations—particularly those with *P* values near the *α*=.05 threshold (eg, *P*=.046 to .049)—should be interpreted as exploratory. These preliminary results are meant to inform the design, targeting, and analytic strategy of future adequately powered mechanistic trials, not to establish efficacy or definitive effect sizes.

Fourth, the single-arm design precludes causal inference and limits our ability to distinguish natural recovery from VR-specific effects. In this pre-post feasibility pilot, all participants received the VR intervention, making it unclear whether observed neural and behavioral changes reflect VR-related processes, spontaneous recovery following subacute musculoskeletal injury, or their interaction. Given the bidirectional relationship between cognitive-affective processes and prefrontal coactivation, it is also possible that neural and psychological changes emerged in parallel rather than through a unidirectional, treatment-driven mechanism. In the absence of a control condition, improvements in pain outcomes and putative mechanisms cannot be attributed specifically to VR and may reflect confounds commonly identified in pain research, including regression to the mean (eg, elevated pain at baseline that abates over time), expectancy and placebo effects, and nonspecific time and attention associated with study visits and staff interactions [112]. Importantly, isolated acute orthopedic injuries are also typically associated with substantial pain reduction within the first several months following injury or surgery, independent of adjunctive behavioral interventions [113]. Accordingly, all findings are interpreted as pre-post associations within a single cohort rather than evidence of efficacy or VR-specific neuroplasticity. These limitations underscore the necessity of future randomized controlled mechanistic trials incorporating appropriate control conditions to disentangle natural recovery from intervention-specific neural and behavioral effects.

Finally, the fixed VR task order may have introduced order effects [114]. We were constrained by the sequential RelieVRx program and the practical need to maintain optode placement and data quality. We did not observe participant fatigue, as evidenced by the VR task manipulation checks. While similar fixed-order designs appear across fNIRS feasibility and real-world studies [115-117], future studies should incorporate counterbalancing to isolate task-specific neural adaptations. Although tasks and acquisition were standardized and correlations used within-subject change scores, individualized movement amplitudes and residual measurement noise may still contribute variance; results should be viewed as exploratory pending confirmation in larger samples.

### Conclusions

The findings of this study provide proof of concept that VR engagement was associated with measurable pre-post differences in PFC activation and FC, which are associated with improvements in behavioral pain outcomes. The involvement of the PFC in modulating pain through VR also supports investigating whether neuromodulatory targeting of this region could enhance therapeutic effects. Notably, both VR and fNIRS have emerged as complementary technologies with accelerating momentum in neuroscience and rehabilitation research. Their combined application, especially within immersive, ecologically valid environments, enables simultaneous intervention and measurement of cortical dynamics during real-time engagement [43]. This convergence supports the development of closed-loop, precision medicine approaches, such as adaptive VR-based neurofeedback systems that can tailor therapeutic content based on individualized brain–behavior profiles. Future research should include larger, more diverse cohorts, integrate multimodal neuroimaging to validate and extend these findings, and use longitudinal designs to evaluate the durability of VR-induced effects. Investigating interindividual variability in neural response will also be critical for optimizing and personalizing VR-based care.

To address these gaps, we are planning a mechanistic clinical trial of RelieVRx that will examine brain–behavior relationships using whole-head fNIRS and investigate the biomechanical and neural mechanisms underlying rehabilitation following subacute traumatic musculoskeletal injuries. Additional planned studies include a multisite randomized clinical trial to confirm the feasibility of the VR and fNIRS protocol across different orthopedic settings with more diverse patient samples and a subsequent efficacy trial of RelieVRx versus a sham VR control. This program of research will advance the refinement of VR-based therapies toward precision subacute pain management. Such trials, if successful, may advance this approach to individualized medicine.

## Supplementary material

10.2196/77713 Multimedia Appendix 1 CONSORT diagram of study participants.

10.2196/77713 Multimedia Appendix 2 Baseline injury and treatment details.

## Abbreviations

CONSORT-EHEALTH: Consolidated Standards of Reporting Trials of Electronic and Mobile Health Applications and Online Telehealth
dlPFC: dorsolateral prefrontal cortex
DMN: default-mode network
FC: functional coactivation
fNIRS: functional near-infrared spectroscopy
HbO: oxygenated hemoglobin
MD: mean difference
mPFC: medial PFC
PCS: Pain Catastrophizing Scale
PFC: prefrontal cortex
VR: virtual reality
