# Supplementary material for: Virtual Reality–Based Pain Modulation in Subacute Musculoskeletal Injury: Functional Near-Infrared Spectroscopy Study of Neural and Behavioral Correlates
Source: JMIR Serious Games. 2026 Mar 30;14:e77713. doi: 10.2196/77713 (PMC13035087; doi:10.2196/77713)
Supplement: Multimedia Appendix 1 [file games-v14-e77713-s001.pptx]

## Slide 1
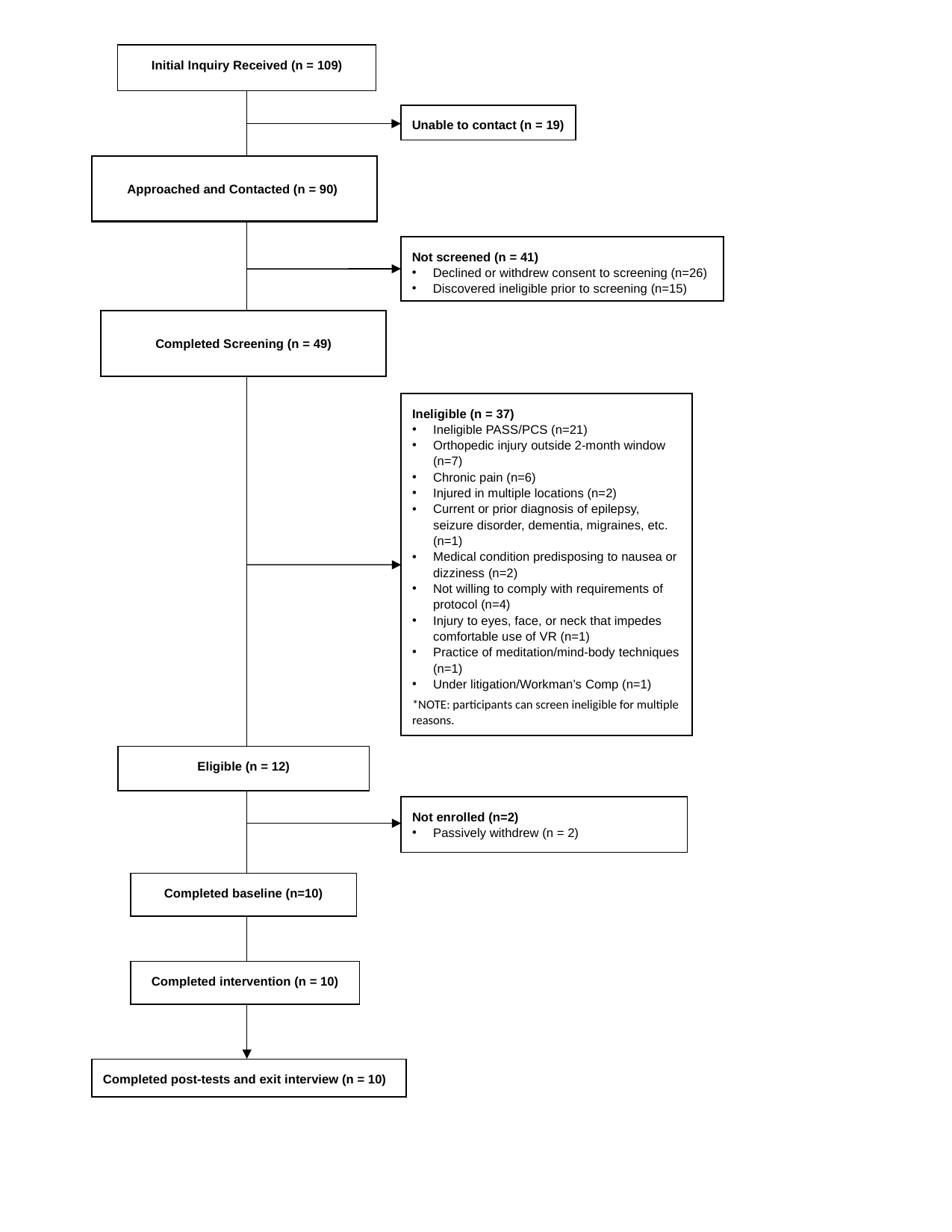

Initial Inquiry Received (n = 109)
Unable to contact (n = 19)
Approached and Contacted (n = 90)
Not screened (n = 41)
Declined or withdrew consent to screening (n=26)
Discovered ineligible prior to screening (n=15)
Completed Screening (n = 49)
Ineligible (n = 37)
Ineligible PASS/PCS (n=21)
Orthopedic injury outside 2-month window (n=7)
Chronic pain (n=6)
Injured in multiple locations (n=2)
Current or prior diagnosis of epilepsy, seizure disorder, dementia, migraines, etc. (n=1)
Medical condition predisposing to nausea or dizziness (n=2)
Not willing to comply with requirements of protocol (n=4)
Injury to eyes, face, or neck that impedes comfortable use of VR (n=1)
Practice of meditation/mind-body techniques (n=1)
Under litigation/Workman’s Comp (n=1)
*NOTE: participants can screen ineligible for multiple reasons.
Eligible (n = 12)
Not enrolled (n=2)
Passively withdrew (n = 2)
Completed baseline (n=10)
Completed intervention (n = 10)
Completed post-tests and exit interview (n = 10)
