# Supplementary material for: Virtual Reality–Based Pain Modulation in Subacute Musculoskeletal Injury: Functional Near-Infrared Spectroscopy Study of Neural and Behavioral Correlates
Source: JMIR Serious Games. 2026 Mar 30;14:e77713. doi: 10.2196/77713 (PMC13035087; doi:10.2196/77713)
Supplement: Multimedia Appendix 2 [file games-v14-e77713-s002.docx]

### Multimedia Appendix 2

Baseline injury and treatment details. *Note.* Days of pain medication = Number of days/week participants reported taking nonnarcotic pain medications at baseline. All participants were beyond the window for opioid pain medications at the time of enrollment in the study. PT = engagement in concurrent physical therapy at baseline. ORIF = open reduction and internal fixation.

| **ID** | **Injury** | **Injury Mechanism** | **Days since injury** | **Surgery** | **Days since surgery** | **PT** | **Days of pain medication use** |
| --- | --- | --- | --- | --- | --- | --- | --- |
| 1 | Nondisplaced fracture of left fifth metatarsal bone | Fall | 17 | N/A | N/A | Yes | 3 |
| 2 | Closed fracture of right tibia and fibula at the pilon | Fall | 32 | ORIF Fracture tibia | 31 | No | 3 |
| 3 | Closed fracture of upper end of left humerus (Left distal radius fracture) | Motor vehicle accident | 138 | ORIF Fracture humerus | 51 | Yes | 3 |
| 4 | Right wrist fracture | Fall | 31 | N/A | N/A | Yes | 0 |
| 5 | Bimalleolar, closed right ankle fracture | Sports injury | 17 | ORIF Fracture ankle | 9 | Yes | 7 |
| 6 | Rupture of ulnar collateral ligament of left first finger; Subluxation of metacarpophalangeal joint of left first finger | Hit by object | 17 | Repair ligament finger surgery | Enrolled 3 days prior to surgery | No | 0 |
| 7 | Closed displaced fracture of proximal end of right humerus | Fall | 24 | N/A | N/A | No | 0 |
| 8 | Displaced bimalleolar fracture of shaft of left fibula | Fall | 24 | ORIF fracture nonunion ankle | 16 | Yes | 0 |
| 9 | Closed displaced fracture of distal phalanx of left fourth finger | Fall | 47 | ORIF fracture finger distal phalanx - left fourth finger; Repair tendon finger – left fourth finger | 36 | Yes | 2 |
| 10 | Closed displaced fracture of intermediate phalanx of left fifth finger | Sports injury | 37 | ORIF fracture finger – left fifth finger with allograft | 20 | No | 1 |
